# Virtual reality-based exposure with applied biofeedback for social anxiety disorder

**DOI:** 10.1192/j.eurpsy.2021.486

**Published:** 2021-08-13

**Authors:** M. Ernst, M. Lichtenstein, L. Clemmensen, T. Andersen, S. Bouchard

**Affiliations:** 1 Department Of Clinical Research, University of Southern Denmark, Odense C, Denmark; 2 Research Unit For Telepsychiatry And E-mental Health, Centre For Telepsychiatry, Mental Health Services in the Region of Southern Denmark, Odense, Denmark; 3 Research Unit For Telepsychiatry And E-mental Health., Centre for Telepsychiatry in the Mental Health Services in the Region of Southern Denmark., Odense, Denmark; 4 Department Of Psychology, University of Southern Denmark, Odense, Denmark; 5 Department Of Psychoeducation And Psychology, University du Québec en Outaouais, quebec, Canada

**Keywords:** virtual reality, social anxiety disorder, Biofeedback, exposure

## Abstract

**Introduction:**

Social Anxiety Disorder (SAD) is considered the most prevalent anxiety disorder with the highest disease burden amongst anxiety disorders. Despite available effective treatment with Cognitive Behavioral Therapy, a majority of individuals with SAD do not seek treatment and many drop out when confronted with elements of exposure. Several studies highlight the many advantages virtual reality exposure holds over in vivo exposure. In this study, we investigate the added effect of real-time biofeedback during virtual reality exposure.

**Objectives:**

The current study is part of a large scale study called VR8. The current study aims to develop and evaluate the feasibility of a VR-biofeedback-intervention for adults with mild to severe social anxiety disorder, before continuing randomized controlled trials.

**Methods:**

Data from semi-structured interviews and surveys will be compared to biodata collected during VR exposure. Participants include a minimum of (n=10) patients and (n=10) clinicians from the Mental Health Services in the Region of Southern Denmark. Surveys include questionnaires used for assessment of anxiety symptoms, usability of technology, and presence in the virtual environment. Collected biodata includes heart rate variability and electrodermal activity. Behavioral markers include eye-gaze. The findings will be analyzed and discussed in a mixed methods design.

**Results:**

The study is ongoing. Preliminary results will be available at presentation.

**Conclusions:**

Successful development and implementation of a biofeedback-informed virtual reality exposure intervention may provide increased reach for patients and individuals who would have otherwise not sought- or dropped out of regular treatment, as well as inform the clinician on how to proceed during virtual exposure.

**Conflict of interest:**

Prof. Stephané Bouchard is consultant to and own equity in Cliniques et Développement In Virtuo, which develops virtual environments, and conflicts of interests are managed according to UQO’s conflict of interests policy; however, Cliniques et Développeme

